# Influenza transmission during COVID-19 measures downscaling in Greece, August 2022: evidence for the need of continuous integrated surveillance of respiratory viruses

**DOI:** 10.2807/1560-7917.ES.2023.28.28.2200754

**Published:** 2023-07-13

**Authors:** Kassiani Mellou, Kyriaki Tryfinopoulou, Mary Emmanouil, Kassiani Gkolfinopoulou, Spyros Sapounas, Maria Evangelidou, Panagiota Moulopoulou, Eleftherios Miaoulis, Emmanouil Angelakis, George Sourvinos, Theoklis Zaoutis, Dimitrios Paraskevis

**Affiliations:** 1 National Public Health Organization, Athens, Greece; 2 Diagnostic Department and Public Health Laboratories, Hellenic Pasteur Institute, Athens, Greece; 3 Laboratory of Clinical Virology, School of Medicine, University of Crete, Heraklion, Greece

**Keywords:** Influenza, co-infections, vaccination

## Abstract

After the near absence of influenza and other respiratory viruses during the first 2 years of the COVID-19 pandemic, an increased activity of mainly influenza A(H3N2) was detected at the beginning of August 2022 in Greece on three islands. Of 33 cases with respiratory symptoms testing negative for SARS-CoV-2 with rapid antigen tests, 24 were positive for influenza: 20 as A(H3N2) subtype and four as A(H1N1)pdm09 subtype. Phylogenetic analysis of selected samples from both subtypes was performed and they fell into clusters within subclades that included the 2022/23 vaccine strains. Our data suggest that influenza can be transmitted even in the presence of another highly infectious pathogen, such as SARS-CoV-2, with a similar transmission mode. We highlight the need for implementing changes in the current influenza surveillance and suggest a move from seasonal to continuous surveillance, especially in areas with a high number of tourists. Year-round surveillance would allow for a timelier start of vaccination campaigns and antiviral drugs procurement processes.

**Key public health message**

**What did you want to address in this study?**

The COVID‐19 pandemic strongly impacted on the circulation of influenza viruses. However, since the relaxation of restrictions, we identified influenza cases in touristic locations in Greece during August 2022. We wanted to investigate this unusual out-of-season occurrence and identify appropriate public health actions, from both a surveillance and response perspective.

**What have we learnt from this study?**

The increased number of people with influenza and other respiratory illnesses on the Greek islands, found outside the influenza surveillance period starting in week 40 and ending in week 20 in the following year, highlights a need to monitor respiratory viruses throughout the year in the post-COVID-19 pandemic era.

**What are the implications of your findings for public health?**

Continuous monitoring for influenza may be needed in patients with respiratory symptoms during periods that have typically had only a low number of influenza cases, especially in areas with tourists coming from both hemispheres. Influenza vaccination campaigns and antiviral drugs procurement should be timely.

## Background

Influenza is a highly contagious respiratory illness caused by influenza viruses, which leads to annual epidemics and, occasionally, pandemics [1]. Each year, influenza affects millions of people worldwide and results in surges of medical visits and increases in healthcare utilisation [2]. In temperate climates in the northern hemisphere, seasonal epidemics occur mainly during winter and the typical influenza surveillance period starts at week 40 and ends at week 20, while in tropical regions, influenza may occur throughout the year, causing outbreaks more irregularly [3]. Vaccination against influenza virus is one of the most effective methods for preventing and controlling virus spread and can notably reduce the incidence of influenza virus infection and related medical complications. The efficacy varies by influenza strain and vaccine match in a given year [4].

Influenza surveillance in Greece is based on primary healthcare sentinel physicians who record the number of cases with influenza-like illness (ILI) (fever > 38 °C and at least one symptom from the upper respiratory system) along with the number of consultations from all causes on a weekly basis. The system was established in 2004, enabling weekly ILI rates monitoring throughout the year. In addition, from week 40 to week 20, clinical specimens are collected every week from a subset of ILI patients and tested for influenza viruses. Since spring 2020, testing for SARS-CoV-2 was also added along with testing for influenza. In the non-seasonal influenza period, patients recorded with ILI are not routinely laboratory tested for the detection of the viruses.

The influenza activity during 2020/21 and 2021/22 was notably low worldwide, presumably because of the measures taken to control the COVID-19 pandemic, such as restrictions on travel and mass gatherings, school closures and measures of physical distancing and mask wearing [5].

### Outbreak detection

At the beginning of August 2022, the Hellenic National Public Health Organisation (NPHO) received notifications about cases with respiratory symptoms who had tested negative for COVID-19 by rapid antigen test at the health care units of three popular touristic islands of the island group of Cyclades in the Central Aegean Sea, south-east of mainland Greece. Cases appeared not to be epidemiologically linked with a specific event or area of the islands. However, there were reports of small clusters of cases, especially among young people travelling together, mostly high school graduates and students. Although not all cases were laboratory-tested because of the limited testing capacity on the islands, there were reports of type A influenza cases among travellers returning to Athens from holidays on these islands. Given the unusual situation of possible increased circulation of influenza virus during summertime, the National Public Health Organization along with the Influenza Reference Laboratory for Southern Greece of the Hellenic Pasteur Institute, decided to review available data and intensify laboratory testing.

Here, we summarise the results of an ad hoc epidemiological and laboratory investigation of a possible increased number of influenza cases in August in a touristic area of Greece and identify surveillance gaps for appropriate public health actions to be considered.

## Methods

### Ad hoc collection of respiratory samples

The primary healthcare centres on the three islands in the Cyclades are public healthcare units that operate on a 24 h/7 day-per-week basis to provide medical services for both residents and tourists. The directors of the three centres confirmed that several cases with respiratory symptoms in the beginning of August 2022 had tested negative for SARS-CoV-2. This was noted as unusual and in contrast to the 2021 tourist season, during which SARS-CoV-2 infection indisputably prevailed among patients with respiratory symptoms visiting the centres. Given this information, our team at the Directorate of Epidemiological Surveillance and Response for Infectious diseases, asked the healthcare centres to collect 10–15 nasopharyngeal swabs at random from patients presenting with a clinical picture compatible with a respiratory infection. Swabs were sent for laboratory investigation to the Central Public Health Laboratory (CPHL) in Athens. Considering the very high workload of the three centres and the absence of historical surveillance data, no retrospective or prospective collection of surveillance data was requested.

As the harbours in the Attica region are the main point of travel for tourists to/from the Cyclades islands, a healthcare centre located closest to one of the harbours was contacted to query about cases. The director of the centre confirmed an increased number of cases with respiratory symptoms among travellers returning from the three islands. Random sampling from five to 10 cases from this centre was also requested.

### Laboratory investigation

#### Initial syndromic testing

Samples were initially analysed at the CPHL using the BioFire Filmarray Respiratory Panel 2.1 plus (BioFire Diagnostics, Salt Lake City, United States (US), which allowed the molecular detection of 22 pathogens (18 viral, SARS-CoV-2 included, and 4 bacterial) within a single specimen.

#### Characterisation of SARS-CoV-2 and influenza strains

Subsequent real-time RT-PCR was performed for samples positive for COVID-19 at the CPHL and for influenza at the Hellenic Pasteur Institute for further characterisation. Both centres are in the Attica region in Greece.

For SARS-CoV-2 detection, an initial pre-screening protocol using the TaqPath COVID-19 CE-IVD RT-PCR kit (ThermoFisher Scientific) was used, which has been shown to detect the deletion of nucleotides 207–212 (Δ69/70) in the S gene of SARS-CoV-2. This deletion results in S gene target failure (SGTF), indicative of one of the SARS-CoV-2 Omicron subvariants BA.1, BA.4 or BA.5 that circulated since late 2021–early 2022 (BA.1) and since June 2022 (BA.4 and BA.5) in Greece. Further characterisation was performed by whole genome sequencing of the virome on the Illumina NextSeq2000 platform in samples with sufficiently high viral load (quantification cycle (Cq) < 30).

The detection of influenza type A and B viruses and the subtyping of influenza A (H3 or H1pdm09) were performed by real-time one-step RT-PCR with specific primers and probes using the US Centers for Disease Control and Prevention (CDC) influenza real-time RT-PCR kit provided by the International Reagent Resource (https://www.internationalreagentresource.org).

For sequence analysis, a 1,096-bp DNA fragment of the HA1 domain was amplified directly from the clinical samples, as previously described [6]. Primer sequences for PCR and sequencing have been published by World Health Organization (WHO) Country Cooperation Strategy in the European Influenza Surveillance Network (EISN) protocol library, with restricted access among the National Influenza Centres. The resulting amplicons were purified by using the QIAquick PCR purification kit (Qiagen) and were sequenced in both directions. Multiple sequence alignment of the sequences obtained and influenza virus reference strains retrieved from GenBank and the Global Initiative on Sharing All Influenza Data (GISAID) [7] was performed using the BioEdit sequence alignment editor (http://www.mbio.ncsu.edu/bioedit/bioedit.html). The origin of the haemagglutinin sequences of influenza A(H1N1)pdm09 and influenza A(H3N2) isolates used for this study are provided in Supplementary Tables S1 and S2, respectively. 

#### Phylogenetic analysis of influenza strains

Phylogenetic analysis was performed using neighbour-joining method with Kimura two-parameter substitution model, as implemented in the Molecular Evolutionary Genetics Analysis (MEGA) software package (version 7). Tree accuracy was tested by bootstrap analysis with 1,000 replicates.

## Results

In total, we tested 33 samples from 33 symptomatic individuals who visited one of three Cycladic islands or the healthcare centre located near the harbour in Attica in August 2022. The median age of the cases was 19 years (interquartile range: 18–23), of whom 20 were men and 28 were of Greek nationality.

### Initial syndromic investigation

Twenty-nine samples were positive for at least one respiratory viral pathogen. Of these 29, 16 samples were positive for one pathogen, nine for two pathogens, and four for more than two pathogens. Four samples tested negative for all 22 respiratory pathogens of the panel. Overall, 25 samples were found positive for influenza: 18 for the A(H3N2) subtype, three for the A(H1N1)pdm09 subtype, and four samples for both subtypes. Influenza H3N2 was the most frequently detected pathogen (n = 22 positive samples) (Table 1).

**Table 1:** Results of syndromic investigation of cases reporting respiratory illness from three Cycladic islands, Greece, August 2022 (n = 33)

Place of sampling	Samples collected	Positive samples				Negative samples
		Influenza Α(Η3Ν2)	Influenza Α(Η1Ν1)pdm09	SARS-CoV-2	Other pathogen^a^	
Island 1	15	9	1	1	4	2
Island 2	5	5	0	3	0	0
Island 3	11	7	6	2	5	1
Healthcare centre (Attica harbour)	2	1	0	1	0	1
**Total**	33	22	7	7	9	4

SARS-CoV-2: severe acute respiratory syndrome coronavirus 2.

^a^ Other pathogens include: rhinovirus/enterovirus, parainfluenza virus 4 and human metapneumovirus.

Co-infections (detection of at least two pathogens) were identified in 13 patients. Of note, SARS-CoV-2 was detected in seven samples that were also positive for influenza: six with the A(H3N2) subtype and one with the A(H1N1)pdm09 subtype. Other co-circulating respiratory viruses were also detected, such as rhinovirus/enterovirus, parainfluenza virus 4 and human metapneumovirus in various combinations with influenza. Table 2 summarises the co-infections detected in 12 influenza A(H3N2) subtype cases.

**Table 2:** Results of syndromic investigation for samples positive for influenza A(H3N2) and one or more other pathogens, three Cycladic islands, Greece, August 2022 (n = 12)

Place of sampling	Samples positive for influenza Α(Η3Ν2) and one or more other pathogens (n/N)	Pathogen (number of co-infections detected)
Island 1	2/9	SARS-CoV-2 (n = 1) Rhinovirus/enterovirus and human metapneumovirus (n = 1)
Island 2	3/5	SARS-CoV-2 (n = 3)
Island 3	6/7	Influenza Α (Η1Ν1)pdm09 (n = 2) Rhinovirus/enterovirus (n = 1) Influenza Α (Η1Ν1pdm09), rhinovirus/enterovirus and parainfluenza virus 4 (n = 1) Influenza Α (Η1Ν1pdm09) and parainfluenza virus 2 (n = 1) SARS-CoV-2 and rhinovirus/enterovirus (n = 1)
Healthcare centre (Attica harbour)	1/1	SARS-CoV-2 (n = 1)

SARS-CoV-2: severe acute respiratory syndrome coronavirus 2.

### Characterisation of SARS-CoV-2 and influenza strains

Amplification of the SARS-CoV-2 viral genome by RT-PCR was successful only in four of seven samples positive by the Filmarray assay (Cq < 38), presenting an SGTF pattern. Whole genome sequencing analysis was technically possible in one isolate, which was classified within the Omicron BA.5 lineage.

Twenty-four of the 25 samples positive for influenza by the syndromic test were confirmed by real-time RT-PCR at the NIC: 20 as A(H3N2) subtype and four as A(H1N1)pdm09 subtype.

### Phylogenetic analysis

Ten samples were selected for phylogenetic analysis, depending on the viral load (Cq < 30 and the island of origin: six were influenza A(H3N2) and four were influenza A(H1N1)pdm09.

**Figure 1:**
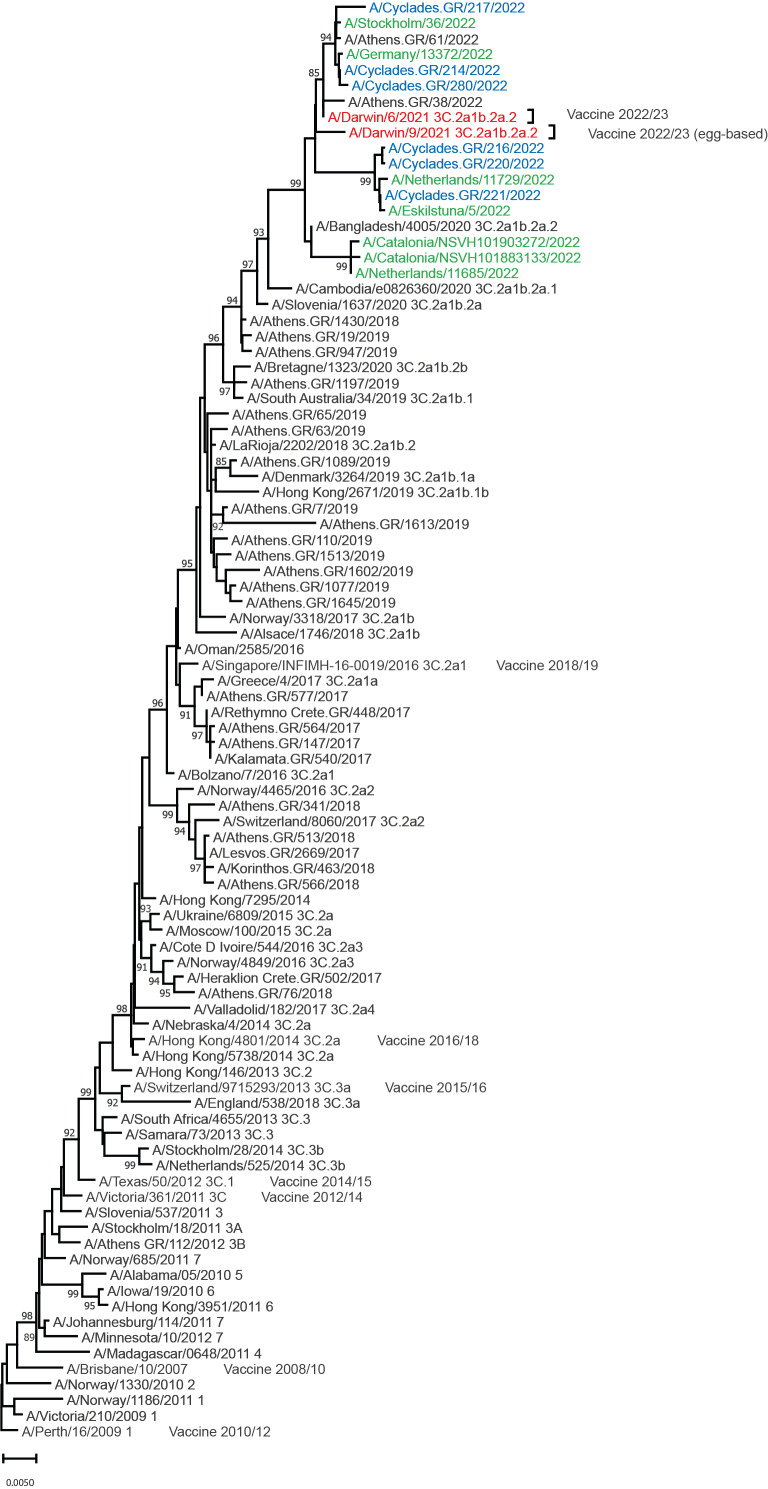
Phylogenetic comparison of influenza A(H3N2) haemagglutinin gene sequences from three Cycladic islands, Greece, August 2022 (n = 6) and haemagglutinin reference and vaccine strain sequences, available up to August 2022 (n = 86)

Phylogenetic analysis of A(H3N2) viruses was performed on haemagglutinin gene sequences and all six strains fell within subclade 3C.2a1b.2a.2 (Figure 1). The A/Darwin/6/2021 and A/Darwin/9/2021 strains, which were included in the northern hemisphere vaccine for 2022/23 season also fell in this clade. The A(H3N2) virus strains derived from Sweden, Germany, the Netherlands, and Spain that circulated during this period [8] were included in the phylogenetic analysis (Figure 1).

Phylogenetic analysis was conducted on reported influenza haemagglutinin reference sequences downloaded from the EpiFlu database of GISAID. Colour coding indicates the type A(H3N2) northern hemisphere 2022/23 vaccine strains in red (n = 2), reference strains in black (n = 77), influenza cases from the three Greek Cycladic islands in blue (n = 6) and influenza cases from other European countries in green (n = 7). Within the group of reference strains in black, vaccine strains dating from 2008 to 2019 are indicated next to the respective strain.

**Figure 2:**
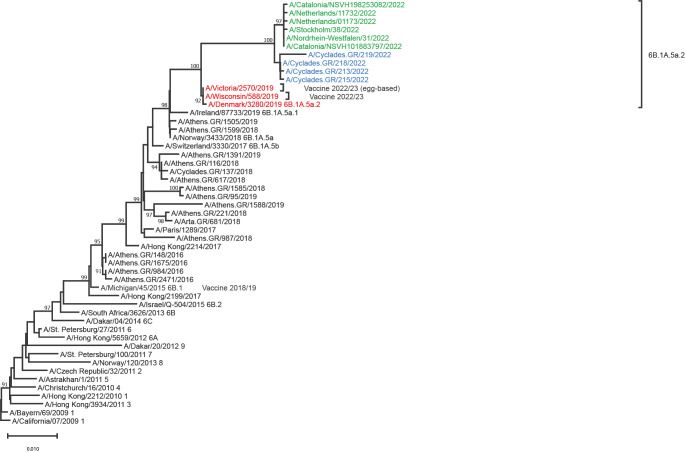
Phylogenetic comparison of influenza A(H1N1) haemagglutinin gene sequences from three Cycladic islands, Greece, August 2022 (n = 4) and haemagglutinin reference and vaccine strain sequences, available up to August 2022 (n = 47)

Phylogenetic analysis of A(H1N1)pdm09 viruses was performed on haemagglutinin gene sequences and all four strains belonged to subclade 6b.1a.5a.2 (Figure 2). The A/Victoria/2570/2019 and A/Wisconsin/588/2019 strains, which were included in the northern hemisphere vaccine for 2022/23 were found in the same clade. A(H1N1) virus strains derived from Sweden, Germany, the Netherlands, and Spain circulating late summer months [8] were compared phylogenetically to current strains from the Greek islands and fell into the same clades (Figure 2).

Phylogenetic analysis was conducted on reported influenza haemagglutinin reference sequences downloaded from the EpiFlu database of GISAID. Colour coding indicates the type A(H1N1) northern hemisphere 2022/23 vaccine strains in red (n = 3), reference strains in black (n = 38), influenza cases from the three Greek Cycladic islands in blue (n = 4) and influenza cases from other European countries in green (n = 6). Within the group of reference strains in black, the vaccine strain of the period 2018/19 is indicated next to the respective strain. Respective clades are indicated next to the branches.

### Outbreak control measures

Following the initial signal for the possible increased activity of influenza on the islands, we communicated to all health care units of the country about the importance of staying alert for diagnosis and reporting of influenza cases during the summer period. Furthermore, we issued a press release and started to produce weekly situation updates for the Ministry of Health. Finally, at the end of the summer, an ad hoc report summarizing influenza epidemiological data was published on 5 September on the website of the Hellenic National Public Health Organization (EODY) (https://eody.gov.gr/enimerosi-schetika-me-kroysmata-gripis-dedomena-apo-to-systima-ypochreotikis-dilosis-nosimaton-ioylios-aygoystos-2022).

The importance of implementing generic prevention measures for respiratory diseases was stressed to the public, focusing on hygiene measures, avoiding contact with symptomatic persons, and abstaining from social activities if experiencing symptoms. Furthermore, a general recommendation for young people to avoid meeting with parents and grandparents after their return from holidays, and especially if experiencing symptoms, was given.

By the middle of September, influenza activity returned to normal levels, whereas no severe cases among the older people (> 65 years) were notified.

## Discussion

Our findings showed a notable activity of influenza A(H3N2) in August 2022 in Greece, localised on three popular touristic islands of the Cyclades.

Interestingly, during this period, influenza activity remained low in the United Kingdom (UK) and the US [9,10], while in the WHO European Region, influenza activity reached levels well above those observed in the 2020/21 influenza season [8]. This was reflected both in terms of geographic spread, i.e. the geographic distribution of reported detections of influenza viruses in specimens, and intensity, i.e. measure of influenza activity, considering both weekly ILI or acute respiratory infection (ARI) rates compared with previous seasons, as well as influenza virus detections.

In particular, European Centre for Disease Prevention and Control (ECDC) data based on countries’ systematic weekly reporting showed that in weeks 31–35 in 2022 (26 July–4 September 2022), there were European countries that reported local (Malta), regional (Scotland) and even wide (Portugal) geographic spread. Low level influenza activity was also reported in Azerbaijan, Georgia, Luxembourg and Slovenia. In the same period, influenza positivity peaked at 10% in Israel and 13% in Spain in sentinel primary care samples [8]. In addition to reports from Europe, data from the Chinese National Influenza Centre also noted markedly higher ILI cases recorded in July 2022 compared with the same period in previous years [11].

This increase of influenza out of the regular influenza season in the northern hemisphere may have several possible explanations. On one hand, this increase may have been due to increased sensitisation of healthcare and the community for respiratory symptoms because of the COVID-19 pandemic; the identification of patients with respiratory symptoms who were negative for SARS-CoV-2 with rapid tests prompted clinicians to report unusual increases of non-COVID-19 cases in the community. In addition, the availability of rapid tests also increased laboratory capacity for the diagnosis of SARS-CoV-2 infection on an unprecedented scale, especially in remote or secluded areas, such as the islands [12,13]. On the other hand, hypotheses on the occurrence of an unusual number of cases of other infectious diseases recently, like the acute hepatitis cases of unknown origin in children have been linked to the pandemic and the non-pharmaceutical measures taken to control it, with increased susceptibility of the population to pathogens [14,15].

Some studies have suggested that the implementation of strict measures of isolation, physical distancing and mask-wearing, which led to a low circulation of respiratory viruses for two consecutive years, may have had an impact on the level of immunity and immune response of the general population [16,17]. Immunity against influenza acquired through natural infection lasts approximately 8–9 months for non-vulnerable populations. Therefore, the absence of exposure in the last years may have attenuated humoral immunity [18].

Overall, the COVID‐19 pandemic seems to have strongly impacted the epidemiology of influenza in particular. In Europe, New Zealand, the US and Western Australia, a decrease of 90% or more in the detection of influenza virus infections was reported in the years 2020 to 2022 compared to the previous winter seasons in the respective hemispheres [5,19,20]. Another factor that could explain the notable out-of-season activity of influenza on the three islands might be that the number of tourists that visited Greece that summer was high, similar to the pre-pandemic levels. After 2 years of travel restrictions, Greece received large numbers of tourists with increased interest in travelling and seeking tourism services. Even though most recorded cases were Greek residents, the existence of cases among tourists cannot be ruled out, as many of the cases may have been treated locally on the islands and then travelled back to their country without a diagnosis. However, there were no reports via the European Union's Early Warning and Response System (EWRS) for travellers diagnosed with influenza after visiting Greece during summer 2022.

As indicated by the phylogenetic analysis of A(H3N2) and A(H1N1) viruses circulating during the late summer months of 2022 in several European countries, the viruses detected belonged to clades already circulating the previous 2 years. Representative strains of A(H3N2) clade 3c.2a1b.2a.2 were also included in the WHO recommendation for the 2022/23 influenza vaccine indicating the right direction for the upcoming 2022/23 winter season. Same is the case for A(H1N1) strains of 6B.1A.5a.2 clade that were also going along with the 2022/23 vaccine recommendations. 

Cases may have originated either indirectly from visitors who were infected in their home countries by people from the southern hemisphere or directly from tourists from the southern hemisphere who have not been recorded, although there is no concrete evidence available. 

The fact that the three islands are popular destinations, especially for younger people, mostly secondary school graduates and students, probably also played a role in the occurrence of a high number of respiratory infections, as young people tend to have a higher number of contacts during their summer holidays and to participate in social activities in crowded places, such as beach bars, bars and clubs. 

The high proportion of co-infections recorded during August 2022 suggests that several pathogens were co-circulating on these islands. There have been reports in the literature about co-infections of influenza viruses and SARS-CoV-2. In a systematic review on the burden of co-infections in patients with confirmed SARS-CoV-2 infection, 3% of patients hospitalised with COVID-19 were also co-infected with another respiratory virus; respiratory syncytial virus (RSV) and influenza A being the most common viral pathogens identified [21]. In the UK, among 6,975 patients with SARS-CoV-2, viral co-infection was detected in 583 (8.4%) patients: 227 patients had influenza viruses, 220 patients had respiratory syncytial virus and 136 patients had adenoviruses [22]. Influenza can be transmitted even in the presence of another highly infectious respiratory pathogen, a finding further supported by the epidemiological data of seasonal influenza. In Australia, the number of influenza cases in 2022 was the highest in the past 5 years, starting and peaking earlier in the season while COVID-19 cases remained high [23]. A similar picture has already been documented in Europe [8]. However, this may have been a result of increased laboratory testing because of the pandemic.

By the middle of September, influenza activity returned to normal. This may have been the result of the touristic season ending or of the measures recommended. After the long period of strict measures during the COVID-19 pandemic, a culture of prevention was instilled in the public. However, it is possible that when individuals tested negative for SARS-CoV-2, they were perhaps less inclined to conform with measures that were primarily directed to preventing COVID-19. 

The increased number of cases with respiratory illness described in our study highlights a need for continuous integrated surveillance of respiratory viruses throughout the year in the post-pandemic era, especially in touristic areas of the country not included in the mainland given the various logistic challenges involved, e.g. transportation of the samples. Irregularities in the influenza activity in the post-pandemic era should be anticipated, and year-round surveillance analogous to the tropical regions where outbreaks may occur throughout the year might be considered for European countries. However, this alteration in surveillance should take into consideration the geography and population movement patterns of each country.

## Conclusions

We highlight the need for implementing changes in the current influenza surveillance and suggest a move from seasonal to continuous surveillance, especially in areas with a high number of tourists from both hemispheres. Rapid point-of-care tests for influenza are currently widely available, cheap and accurate, and can be used on the islands throughout the year. Year-round surveillance would allow for a timelier start of vaccination campaigns and antiviral drugs procurement processes when needed, as the protection of the most vulnerable population for developing severe disease, such as those who are older or immunocompromised, is our main public health priority. 

## Supplementary Data

288000 Supplement
