# Supplementary material for: Influenza transmission during COVID-19 measures downscaling in Greece, August 2022: evidence for the need of continuous integrated surveillance of respiratory viruses
Source: Euro Surveill. 2023 Jul 13;28(28):2200754. doi: 10.2807/1560-7917.ES.2023.28.28.2200754 (PMC10347892; doi:10.2807/1560-7917.ES.2023.28.28.2200754)
Supplement: Supplement [file 22-00754_TRYFINOPOULOU_Supplement.pdf]

"This supplementary material is hosted by *Eurosurveillance* as supporting information alongside the article [*Influenza transmission during COVID-19 measures downscaling in Greece, August 2022: evidence for the need of continuous integrated surveillance of respiratory viruses*], on behalf of the authors, who remain responsible for the accuracy and appropriateness of the content. The same standards for ethics, copyright, attributions and permissions as for the article apply. Supplements are not edited by *Eurosurveillance* and the journal is not responsible for the maintenance of any links or email addresses provided therein."

Supplementary Table S1

Origin of the haemagglutinin sequences of influenza A(H1N1)pdm09 isolates used for the phylogenetic analysis as depicted in the corresponding phylogenetic tree in ascending order

Segment ID	Isolate Name	Originating Laboratory	Country	Submitting Laboratory
EPI177294	A/California/07/2009	Naval Health Research Center (NHRC) U.S. Navy	United States	Centers for Disease Control and Prevention
EPI239631	A/Bayern/69/2009		Germany	Centers for Disease Control and Prevention
EPI326206	A/Hong Kong/3934/2011	Government Virus Unit	Hong Kong	National Institute for Medical Research
EPI279895	A/Hong Kong/2212/2010	Government Virus Unit	Hong Kong	National Institute for Medical Research
EPI280344	A/Christchurch/16/2010	WHO Collaborating Centre for Reference and Research on Influenza	New Zealand Australia	Centers for Disease Control and Prevention
EPI319590	A/Astrakhan/1/2011	WHO National Influenza Centre Russian Federation	Russian Federation	National Institute for Medical Research
EPI319447	A/Czech Republic/32/2011	National Institute of Public Health	Czech Republic	National Institute for Medical Research
EPI416411	A/Norway/120/2013	WHO National Influenza Centre	Norway	National Institute for Medical Research
EPI316435	A/St. Petersburg/100/2011	Russian Academy of Medical Sciences	Russian Federation	Centers for Disease Control and Prevention
EPI417122	A/Dakar/20/2012	Institut Pasteur de Dakar	Senegal	National Institute for Medical Research
EPI382424	A/Hong Kong/5659/2012	Hong Kong Department of Health	Hong Kong	Hong Kong Department of Health

EPI319527	A/St. Petersburg/27/2011	WHO National Influenza Centre Russian Federation	Russian Federation	National Institute for Medical Research
EPI539474	A/Dakar/04/2014	Institut Pasteur de Dakar	Senegal	National Institute for Medical Research
EPI466626	A/South Africa/3626/2013	National Institute for Communicable Disease	South Africa	National Institute for Medical Research
EPI697729	A/Israel/Q-504/2015	Central Virology Laboratory Israel (NIC)	Israel	Crick Worldwide Influenza Centre
EPI1082164	A/Hong Kong/2199/2017	Government Virus Unit	Hong Kong	Crick Worldwide Influenza Centre
EPI662594	A/Michigan/45/2015	Michigan Department of Community Health	United States	Centers for Disease Control and Prevention
EPI715800	A/Athens/2471/2016	Hellenic Pasteur Institute	Greece	Hellenic Pasteur Institute
EPI711188	A/Athens.GR/984/2016	Hellenic Pasteur Institute	Greece	Hellenic Pasteur Institute
EPI715794	A/Athens.GR/1675/2016	Hellenic Pasteur Institute	Greece	Hellenic Pasteur Institute
EPI711168	A/Athens.GR/148/2016	Hellenic Pasteur Institute	Greece	Hellenic Pasteur Institute
EPI1019935	A/Hong Kong/2214/2017	Government Virus Unit	Hong Kong	Crick Worldwide Influenza Centre
EPI1215576	A/Attica.GRC/987/2018	Hellenic Pasteur Institute	Greece	Hellenic Pasteur Institute
EPI1043315	A/Paris/1289/2017	Institut Pasteur	France	Institut Pasteur
EPI1241460	A/Arta.GR/681/2018	Hellenic Pasteur Institute	Greece	Crick Worldwide Influenza Centre
EPI1190075	A/Attica.GRC/221/2018	Hellenic Pasteur Institute	Greece	Hellenic Pasteur Institute
EPI1589427	A/Athens.GR/1588/2019	Hellenic Pasteur Institute	Greece	Hellenic Pasteur Institute
EPI1358831	A/Athens.GR/95/2019	Hellenic Pasteur Institute	Greece	Hellenic Pasteur Institute
EPI1352495	A/Athens.GR/1585/2018	Hellenic Pasteur Institute	Greece	Hellenic Pasteur Institute
EPI1226981	A/Attica.GRC/617/2018	Hellenic Pasteur Institute	Greece	Hellenic Pasteur Institute
EPI1190117	A/Kyklades.GRC/137/2018	Hellenic Pasteur Institute	Greece	Hellenic Pasteur Institute
EPI1190121	A/Attica.GRC/116/2018	Hellenic Pasteur Institute	Greece	Hellenic Pasteur Institute
EPI1585540	A/Athens.GR/1391/2019	Hellenic Pasteur Institute	Greece	Hellenic Pasteur Institute
EPI1153822	A/Switzerland/3330/2017	Swiss National Reference Centre for Influenza	Switzerland	Crick Worldwide Influenza Centre
EPI1328929	A/Norway/3433/2018	WHO National Influenza Centre	Norway	Crick Worldwide Influenza Centre
EPI1352504	A/Athens.GR/1599/2018	Hellenic Pasteur Institute	Greece	Hellenic Pasteur Institute
EPI1585553	A/Athens.GR/1505/2019	Hellenic Pasteur Institute	Greece	Hellenic Pasteur Institute
EPI1639187	A/Ireland/87733/2019	UCD National Virus Reference Laboratory	Ireland	UCD National Virus Reference Laboratory

EPI1641015	A/Denmark/3280/2019	Statens Serum Institute	Denmark	Statens Serum Institute
EPI1661758	A/Wisconsin/588/2019	Wisconsin State Laboratory of Hygiene	United States	Wisconsin State Laboratory of Hygiene
EPI2222758	A/Victoria/2570/2019	Alfred Hospital	Australia	WHO Collaborating Centre for Reference and Research on Influenza
EPI2608647	A/Cyclades.GR/215/2022	Hellenic Pasteur Institute	Greece	Hellenic Pasteur Institute
EPI2608646	A/Cyclades.GR/213/2022	Hellenic Pasteur Institute	Greece	Hellenic Pasteur Institute
EPI2608648	A/Cyclades.GR/218/2022	Hellenic Pasteur Institute	Greece	Hellenic Pasteur Institute
EPI2608652	A/Cyclades.GR/219/2022	Hellenic Pasteur Institute	Greece	Hellenic Pasteur Institute
EPI2129785	A/Catalonia/NSVH10188379 7/2022	Hospital Universitari Vall d'Hebron	Spain	Hospital Universitari Vall d'Hebron
EPI2176323	A/Nordrhein-Westfalen/31/2022	Robert Koch-Institute Nationales Referenzzentrum für Influenza	Germany	Robert Koch-Institute Nationales Referenzzentrum für Influenza
EPI2187511	A/Stockholm/38/2022	Klinisk mikrobiologi, Karolinska Universitetslaboratoriet, Karolinska Universitetssjukhuset Solna	Sweden	Public Health Agency of Sweden
EPI2129627	A/Netherlands/01173/2022	Erasmus Medical Center	Netherlands	Erasmus Medical Center
EPI2192956	A/Netherlands/11732/2022	PAMM	Netherlands	National Institute for Public Health and the Environment (RIVM)
EPI2229454	A/Catalonia/NSVH19825308 2/2022	Hospital Universitari Vall d'Hebron	Spain	Crick Worldwide Influenza Centre

Supplementary Table S2

Origin of the haemagglutinin sequences of influenza A(H3N2) isolates used for the phylogenetic analysis as depicted in the corresponding phylogenetic tree in ascending order

Segment ID	Isolate Name	Originating Laboratory	Country	Submitting Laboratory
EPI182941	A/Perth/16/2009	Pathwest QE II Medical Centre	Australia	WHO Collaborating Centre for Reference and Research on Influenza
EPI190148	A/Victoria/210/2009	Victorian Infectious Diseases Reference Laboratory	Australia	WHO Collaborating Centre for Reference and Research on Influenza

EPI326137	A/Norway/1186/2011	Norwegian Institute of Public Health	Norway	National Institute for Medical Research
EPI302231	A/Norway/1330/2010	WHO National Influenza Centre	Norway	National Institute for Medical Research
EPI155962	A/Brisbane/10/2007		Australia	Centers for Disease Control and Prevention
EPI319276	A/Madagascar/0648/2011	Institut Pasteur de Madagascar	Madagascar	National Institute for Medical Research
EPI376512	A/Minnesota/10/2012	Minnesota Department of Health	United States	Centers for Disease Control and Prevention
EPI335734	A/Johannesburg/114/2011	National Institute for Communicable Disease	South Africa	National Institute for Medical Research
EPI319237	A/Hong Kong/3951/2011	Government Virus Unit	Hong Kong	National Institute for Medical Research
EPI302327	A/Iowa/19/2010	Iowa State Hygienic Laboratory	United States	Centers for Disease Control and Prevention
EPI278805	A/Alabama/05/2010	U.S. Air Force School of Aerospace Medicine	United States	Centers for Disease Control and Prevention
EPI326133	A/Norway/685/2011	Norwegian Institute of Public Health	Norway	National Institute for Medical Research
EPI750018	A/Athens.GR/112/2012	Hellenic Pasteur Institute	Greece	Hellenic Pasteur Institute
EPI318272	A/Stockholm/18/2011		Sweden	Public Health Agency of Sweden
EPI335697	A/Slovenia/537/2011	Laboratory for Virology, National Institute of Public Health	Slovenia	National Institute for Medical Research
EPI349103	A/Victoria/361/2011	Melbourne Pathology	Australia	WHO Collaborating Centre for Reference and Research on Influenza
EPI377499	A/Texas/50/2012	Texas Department of State Health Services-Laboratory Services	United States	Centers for Disease Control and Prevention
EPI574644	A/Netherlands/525/2014	National Institute for Public Health and the Environment (RIVM)	Netherlands	National Institute for Medical Research
EPI552698	A/Stockholm/28/2014		Sweden	Public Health Agency of Sweden
EPI460558	A/Samara/73/2013	WHO National Influenza Centre Russian Federation	Russian Federation	National Institute for Medical Research

EPI466802	A/South Africa/4655/2013	National Institute for Communicable Disease	South Africa	National Institute for Medical Research
EPI1242149	A/England/538/2018	UK Health Security Agency - Colindale	United Kingdom	UK Health Security Agency - Colindale
EPI530687	A/Switzerland/9715293/2013	Swiss National Reference Centre for Influenza	Switzerland	National Institute for Medical Research
EPI426061	A/Hong Kong/146/2013	Government Virus Unit	Hong Kong	National Institute for Medical Research
EPI539806	A/Hong Kong/5738/2014	Government Virus Unit	Hong Kong	National Institute for Medical Research
EPI539576	A/Hong Kong/4801/2014	Government Virus Unit	Hong Kong	National Institute for Medical Research
EPI539619	A/Nebraska/4/2014	Centers for Disease Control and Prevention	United States	National Institute for Medical Research
EPI1154884	A/Valladolid/182/2017	Universidad de Valladolid	Spain	Crick Worldwide Influenza Centre
EPI1177325	A/Athens.GR/76/2018	Hellenic Pasteur Institute	Greece	Crick Worldwide Influenza Centre
EPI978968	A/Heraklion_Crete GR/502/2017	Hellenic Pasteur Institute	Greece	Crick Worldwide Influenza Centre
EPI887439	A/Norway/4849/2016	WHO National Influenza Centre	Norway	Crick Worldwide Influenza Centre
EPI781596	A/Cote D'Ivoire/544/2016	Pasteur Institut of Côte d'Ivoire	Cote d'Ivoire	Crick Worldwide Influenza Centre
EPI624575	A/Moscow/100/2015	Ivanovsky Research Institute of Virology RAMS	Russian Federation	Crick Worldwide Influenza Centre
EPI621815	A/Ukraine/6809/2015	Ministry of Health of Ukraine	Ukraine	Crick Worldwide Influenza Centre
EPI551882	A/Hong Kong/7295/2014	Government Virus Unit	Hong Kong	National Institute for Medical Research
EPI1215574	A/Attica.GRC/566/2018	Hellenic Pasteur Institute	Greece	Hellenic Pasteur Institute
EPI1248638	A/Korinthos.GRC/463/2018	Hellenic Pasteur Institute	Greece	Hellenic Pasteur Institute
EPI1203160	A/Lesvos.GRC/2669/2017	Hellenic Pasteur Institute	Greece	Hellenic Pasteur Institute
EPI1203161	A/Attica.GRC/513/2018	Hellenic Pasteur Institute	Greece	Hellenic Pasteur Institute
EPI1154878	A/Switzerland/8060/2017	Swiss National Reference Centre for Influenza	Switzerland	Crick Worldwide Influenza Centre
EPI1230771	A/Athens.GR/341/2018	Hellenic Pasteur Institute	Greece	Crick Worldwide Influenza Centre

EPI864041	A/Norway/4465/2016	Stavanger Universitetssykehus, Avd. for Medisinsk Mikrobiologi	Norway	Norwegian Institute of Public Health
EPI773595	A/Bolzano/7/2016	Istituto Superiore di Sanità	Italy	Crick Worldwide Influenza Centre
EPI1131124	A/Kalamata/540/2017	National Institute for Medical Research	Greece	Centers for Disease Control and Prevention
EPI904025	A/Athens.GR/147/2017	Hellenic Pasteur Institute	Greece	Hellenic Pasteur Institute
EPI978934	A/Athens GR/564/2017	Hellenic Pasteur Institute	Greece	Crick Worldwide Influenza Centre
EPI991705	A/Rethymno_Crete GR/448/2017	Hellenic Pasteur Institute	Greece	Crick Worldwide Influenza Centre
EPI978940	A/Athens GR/577/2017	Hellenic Pasteur Institute	Greece	Crick Worldwide Influenza Centre
EPI967219	A/Greece/4/2017	Aristotelian University of Thessaloniki	Greece	Crick Worldwide Influenza Centre
EPI780183	A/Singapore/INFIMH-16-0019/2016	Ministry of Health, Singapore	Singapore	Ministry of Health, Singapore
EPI769531	A/Oman/2585/2016	Central Public Health Laboratory, Ministry of Health	Oman	Crick Worldwide Influenza Centre
EPI1232425	A/Alsace/1746/2018		France	Institut Pasteur
EPI1140854	A/Norway/3318/2017	WHO National Influenza Centre	Norway	Crick Worldwide Influenza Centre
EPI1589439	A/Athens.GR/1645/2019	Hellenic Pasteur Institute	Greece	Hellenic Pasteur Institute
EPI1589434	A/Athens.GR/1077/2019	Hellenic Pasteur Institute	Greece	Hellenic Pasteur Institute
EPI1589437	A/Athens.GR/1602/2019	Hellenic Pasteur Institute	Greece	Hellenic Pasteur Institute
EPI1585557	A/Athens.GR/1513/2019	Hellenic Pasteur Institute	Greece	Hellenic Pasteur Institute
EPI1359439	A/Athens.GR/110/2019	Hellenic Pasteur Institute	Greece	Hellenic Pasteur Institute
EPI1589438	A/Athens.GR/1613/2019	Hellenic Pasteur Institute	Greece	Hellenic Pasteur Institute
EPI1352510	A/Spata.GR/07/2019	Hellenic Pasteur Institute	Greece	Hellenic Pasteur Institute
EPI1543098	A/Hong Kong/2671/2019	Government Virus Unit	Hong Kong	Crick Worldwide Influenza Centre
EPI1641083	A/Denmark/3264/2019	Statens Serum Institute	Denmark	Statens Serum Institute
EPI1589435	A/Athens.GR/1089/2019	Hellenic Pasteur Institute	Greece	Hellenic Pasteur Institute
EPI1256086	A/LaRioja/2202/2018	Instituto de Salud Carlos III	Spain	Crick Worldwide Influenza Centre
EPI1359235	A/Athens.GR/63/2019	Hellenic Pasteur Institute	Greece	Hellenic Pasteur Institute
EPI1359238	A/Athens.GR/65/2019	Hellenic Pasteur Institute	Greece	Hellenic Pasteur Institute
EPI1631902	A/South Australia/34/2019	WHO Collaborating Centre for Reference and Research on Influenza	Australia	Centers for Disease Control and Prevention
EPI1589436	A/Athens.GR/1197/2019	Hellenic Pasteur Institute	Greece	Hellenic Pasteur Institute

EPI1763587	A/Bretagne/1323/2020	Institut Pasteur	France	Crick Worldwide Influenza Centre
EPI1589433	A/Athens.GR/947/2019	Hellenic Pasteur Institute	Greece	Hellenic Pasteur Institute
EPI1352511	A/Athens.GR/19/2019	Hellenic Pasteur Institute	Greece	Hellenic Pasteur Institute
EPI1327523	A/Athens.GR/1430/2018	Hellenic Pasteur Institute	Greece	Hellenic Pasteur Institute
EPI1735371	A/Slovenia/1637/2020	Laboratory for Virology, National Institute of Public Health	Slovenia	Crick Worldwide Influenza Centre
EPI1837753	A/Cambodia/e0826360/2020	Institute Pasteur du Cambodia	Cambodia	WHO Collaborating Centre for Reference and Research on Influenza
EPI2131539	A/Netherlands/11685/2022	ISALA Ziekenhuizen	Netherlands	National Institute for Public Health and the Environment (RIVM)
EPI2129764	A/Catalonia/NSVH101883133/2022	Hospital Universitari Vall d'Hebron	Spain	Hospital Universitari Vall d'Hebron
EPI2178464	A/Catalonia/NSVH101903272/2022	Hospital Universitari Vall d'Hebron	Spain	Hospital Universitari Vall d'Hebron
EPI1838303	A/Bangladesh/4005/2020	icddr,b International Centre for Diarrhoeal Disease Research, Bangladesh	Bangladesh	Centers for Disease Control and Prevention
EPI2187457	A/Eskilstuna/5/2022	Unilabs Mikrobiologi/Laboratoriemedicin, Mälarsjukhuset	Sweden	Public Health Agency of Sweden
EPI2608644	A/Cyclades.GR/221/2022	Hellenic Pasteur Institute	Greece	Hellenic Pasteur Institute
EPI2190949	A/Netherlands/11729/2022	PAMM	Netherlands	National Institute for Public Health and the Environment (RIVM)
EPI2608643	A/Cyclades.GR/220/2022	Hellenic Pasteur Institute	Greece	Hellenic Pasteur Institute
EPI2608641	A/Cyclades.GR/216/2022	Hellenic Pasteur Institute	Greece	Hellenic Pasteur Institute
EPI1859996	A/Darwin/9/2021	Royal Darwin Hospital	Australia	WHO Collaborating Centre for Reference and Research on Influenza
EPI1857216	A/Darwin/6/2021	Royal Darwin Hospital	Australia	WHO Collaborating Centre for Reference and Research on Influenza
EPI2610695	A/Athens.GR/38/2022	Hellenic Pasteur Institute	Greece	Hellenic Pasteur Institute
EPI2608645	A/Cyclades.GR/280/2022	Hellenic Pasteur Institute	Greece	Hellenic Pasteur Institute
EPI2608632	A/Cyclades.GR/214/2022	Hellenic Pasteur Institute	Greece	Hellenic Pasteur Institute
EPI2133251	A/Germany/13372/2022	U.S. Air Force School of Aerospace Medicine	Germany	U.S. Air Force School of Aerospace Medicine
EPI2610696	A/Athens.GR/61/2022	Hellenic Pasteur Institute	Greece	Hellenic Pasteur Institute

EPI2187487	A/Stockholm/36/2022	Klinisk mikrobiologi, Karolinska Universitetslaboratoriet, Karolinska Universitetssjukhuset Solna	Sweden	Public Health Agency of Sweden
EPI2608642	A/Cyclades.GR/217/2022	Hellenic Pasteur Institute	Greece	Hellenic Pasteur Institute
